# Seedling Resistance to Stem Rust and Molecular Marker Analysis of Resistance Genes in Wheat Cultivars of Yunnan, China

**DOI:** 10.1371/journal.pone.0165640

**Published:** 2016-10-28

**Authors:** Tian Ya Li, Yuan Yin Cao, Xian Xin Wu, Xiao Feng Xu, Wan Lin Wang

**Affiliations:** College of Plant Protection, Shenyang Agricultural University, Shenyang, Liaoning, China; GERMANY

## Abstract

Stem rust is one of the most potentially harmful wheat diseases, but has been effectively controlled in China since 1970s. However, the interest in breeding wheat with durable resistance to stem rust has been renewed with the emergence of Ug99 (TTKSK) virulent to the widely used resistance gene *Sr31*, and by which the wheat stem rust was controlled for 40 years in wheat production area worldwide. Yunnan Province, located on the Southwest border of China, is one of the main wheat growing regions, playing a pivotal role in the wheat stem rust epidemic in China. This study investigated the levels of resistance in key wheat cultivars (lines) of Yunnan Province. In addition, the existence of *Sr25*, *Sr26*, *Sr28*, *Sr31*, *Sr32*, and *Sr38* genes in 119 wheat cultivars was assessed using specific DNA markers. The results indicated that 77 (64.7%) tested wheat varieties showed different levels of resistance to all the tested races of *Puccinia graminis* f. sp. *tritici*. Using molecular markers, we identified the resistance gene *Sr31* in 43 samples; *Sr38* in 10 samples; *Sr28* in 12 samples, and one sample which was resistant against Ug99 (avirulent to *Sr32*). No *Sr25* or *Sr26* (effective against Ug99) was identified in any cultivars tested. Furthermore, 5 out of 119 cultivars tested carried both *Sr31* and *Sr38* and eight contained both *Sr31* and *Sr28*. The results enable the development of appropriate strategies to breed varieties resistant to stem rust.

## Introduction

Stem rust (caused by *Puccinia graminis* Pers. f. sp. *tritici* Eriks. & E. Henn.) is one of the most serious diseases of wheat, worldwide [1, 2]. In China, it has been effectively controlled through the development of resistant cultivars and deployment of effective resistance genes, especially 1B/1R translocation gene *Sr31* in different epidemiological regions since 1970s [3, 4]. However, in 1998, a new race of wheat stem rust pathogen designated as Ug99 (TTKSK), expressing virulence to *Sr31*, was first identified in Uganda [5, 6]. It has spread throughout the major wheat growing regions of Africa such as Ethiopia, Zimbabwe, Mozambique, Kenya, Sudan, Yemen, Egypt, and Tanzania [7, 8]. The variants exhibited stronger virulence and could rapidly spread worldwide. For example, variants with virulence against common stem rust-resistance genes *Sr24*, *Sr38*, and *Sr36* have also been detected [9]. According to the Food and Agriculture Organization (FAO) forecasts, this disease may spread eastward from Iran into countries of Central Asia [10]. TTKSK has been detected in Iran [11] and may soon threaten wheat production in the Indian sub-continent [2, 12]. The spread of Ug99 and its variants to India is a threat to the security of wheat production in China.

Yunnan Province, located on the Southwest border of China, is close to India, increasing the risk of Ug99 contamination [3]. The annual urediospores life cycle of *Pgt* is completed in the region, playing a key role in the large-scale epidemic of wheat stem rust [13]. Therefore, the resistance level of the cultivars in Yunnan province has a direct impact on epidemiology. In addition, because of the rarity of the wheat stem rust since the 1970s, the resistance of wheat cultivars has not been taken seriously. Therefore, due to the imminent risk in Chinese wheat production posed by Ug99, analysis of resistance against stem rust and delineation of the resistance genes in the cultivars (lines) locally are of great significance in evaluation of the risk, because 60 percent of wheat varieties contain *Sr31* in China. It also raises the possibility of crop rotation as well as development of new rust-resistant sources. Wheat protection and breeding of resistant cultivars using conventional methods are time-consuming, intricate, and slow, and are influenced by the environment eventually. Currently, plant breeding is updated using molecular markers. Development of molecular markers has led to efficient methods of plant breeding [14]. Various studies have been conducted to confirm the presence of *Sr* genes in wheat cultivars. A collection of 54 wheat cultivars and 11 breeding lines from South Africa was screened to identify *Sr2*, *Sr24 and Sr31* genes using DNA markers [15]. PCR-based DNA markers were used to check rust resistance genes among the 20 wheat genotypes and 22 markers were amplified [16]. Kokhmetova and Atishova [17] studied the presence of *Sr* genes (*Sr2*, *Sr22*, *Sr24*, *Sr36*, and *Sr46*), which are effective against Ug99 in 88 cultivars of spring soft wheat in Kazakhstan. Haile et al. [18] screened out 30 *Sr* genes using SSR and STS markers in 58 tetraploid wheat accessions of Ethiopia. In China, molecular markers closely linked with *Sr22*, *Sr25*, and *Sr33* are used for the detection of wheat cultivars in Heilongjiang Province, and four cultivars may contain *Sr22* and three probably carry *Sr33* [19–21].

In this study, on the basis of resistance levels to Chinese stem rust in wheat cultivars of Yunnan province, the reported molecular markers closely linked to three major resistance genes *Sr31*, *Sr32*, *Sr38* and another three resistance genes effective against Ug99 races (*Sr25*, *Sr26*, and *Sr28*) were used to assess the prevalence of stem rust resistance in Yunnan wheat cultivars. Breeders may use this information to genetically engineer new and potentially durable combinations of stem rust resistance cultivars.

## Materials and Methods

### Wheat cultivars and near-isogenic lines

A total of 119 tested wheat cultivars including the primary cultivars and reserve lines in Yunnan province were provided by Dr. Mingju Li at Yunnan Provincial Institute of Agricultural Environment and Resources.

Six *Sr* genes were tested: *Sr25*, *Sr26*, *Sr28*, *Sr31*, *Sr32*, and *Sr38*. The near-isogenic lines carrying these resistance genes were provided by Dr. Yue Jin from USDA-ARS, Cereal Disease Laboratory, University of Minnesota.

### *P*. *graminis* f. sp. *tritici* races

The tested *Pgt* races include the dominant 21C3CTHTM and 34MKGSM, and 34C3RTGQM. The differentials of races are composed of three parts: four Stakman’s (Little club, Reliance, Einkorn, and Vernal), five Chinese supplemental differentials (Mianzi 52, Huadong 6, Mini 2761, Orofen, and Rulofen), and five sets of 20 single Sr-gene lines (*Sr5*, *Sr21*, *Sr9e*, *Sr7b*, *Sr11*, *Sr6*, *Sr8a*, *Sr9g*, *Sr36*, *Sr9b*, *Sr30*, *Sr17*, *Sr9a*, *Sr9d*, *Sr10*, *SrTmp*, *Sr24*, *Sr31*, *Sr38*, *and McN*). At present, the races were identified and designated using three parts of differentials by Wheat Disease Laboratory, Shenyang Agricultural University, namely: part 1 uses four of Stakman’s hosts and is given Arabic numerals, such as 21 or 34 etc.; the second part uses 5 Chinese supplemental lines, and was given a ‘C’ plus Arabic numerals, C1 or C2 or C3 etc.; and the third part uses 20 single Sr-gene lines and given five-letter-code as currently used in the United States and many other countries, for examples: HTTTM or RKGQM. The full names of the races and their virulence/avirulence patterns are shown in Table 1 [3]. They were isolated and identified by Wheat Disease Laboratory, Shenyang Agricultural University, China.

**Table 1:** Virulence/avirulence patterns of 3 races of *Puccinia graminis* f. sp. *tritici*.

Race	Ineffective *Sr* genes	Effective *Sr* genes
21C3CTHTM	*6*, *7b*, *8a*, *9a*, *9b*, *9d*, *9f*, *9g*, *10*, *11*, *12*, *13*, *14*, *15*, *16*, *17*, *18*, *24*, *28*, *29*, *34*, *35*, *Tmp*, *McN*	*5*, *9e*, *19*, *20*, *21*, *22*, *23*, *25*, *26*, *27*, *30*, *31*, *32*, *33*, *36*, *37*, *38*, *47*
34MKGSM	*5*, *6*, *7b*, *8a*, *9a*, *9b*, *9d*, *9f*, *9g*, *10*, *12*, *15*, *16*, *20*, *24*, *27*, *28*, *29*, *McN*	*9e*, *11*, *13*, *14*, *17*, *18*, *19*, *21*, *22*, *23*, *25*, *26*, *30*, *31*, *32*, *33*, *34*, *35*, *36*, *37*, *38*, *47*, *Tmp*
34C3RKGQM	*5*, *6*, *7b*, *8a*, *9a*, *9b*, *9d*, *9f*, *9g*, *12*, *16*, *19*, *21*, *23*, *24*, *27*, *28*, *29*, *McN*	*9e*, *10*, *11*, *13*, *14*, *15*, *17*, *18*, *20*, *22*, *25*, *26*, *30*, *31*, *32*, *33*, *34*, *35*, *36*, *37*, *38*, *47*, *Tmp*

### DNA extraction and fragment analysis

DNA was extracted from young leaves of seedlings according to the method described by Lagudah et al. [22]. The DNA quality was determined using 1.2% (w/v) agarose gel. DNA quantification was performed using NanoDrop-1000 version 3.3.1 spectrophotometer. PCR primers were synthesized by Sangon Biotech (http://www.sangon.com/, China). PCR assays were performed according to the published protocols (Table 2). PCR amplifications were carried out in 25 μL volume, including 2.5 μL 10×buffer (Mg^2+^), 0.5 μL 10 mmol·L^-1^ dNTPs, 1 μL 10 μmol·L^-1^ of each primer, 0.2 μL 5 U·μL^-1^ *Taq*-polymerase, and 2 μL 30 ng·μL^-1^ DNA. De-ionized water was used to obtain 25 μL. 1.2% (w/v) agarose gel was used to detect the fragments of target gene. The agarose gels were analyzed by Uvitec cambridge. The incubation time and voltage used for analysis of fragments were 200V and 30 min. All above reagents were provided by Sangon Biotech (http://www.sangon.com/, China). Further, the ddH_2_O and reaction system lacking the template DNA were used as controls. The specific reaction conditions were based on published studies.

**Table 2:** The primers linked to resistance genes *Sr25*, *Sr26*, *Sr28*, *Sr31*, *Sr32* and *Sr38*.

Tagged *Sr* genes	Marker	Size of markers (bp)	Primer sequence	References
*Sr25*	Xwmc221	190	F-5′ACGATAATGCAGCGGGGAAT R-5′ GCTGGGATCAAGGGATCAAT	21
*Sr26*	Sr26#43	207	F-5′AATCGTCCACATTGGCTTCT R-5′ CGCAACAAAATCATGCACTA	24
*Sr28*	Wpt-7004	194	F-5′CTCCCACCAAAACAGCCTAC R-5′ AGATGCGAATGGGCAGTTAG	25
*Sr31*	SCSS30.2_576_	576	F-5′GTCCGACAATACGAACGATT R-5′ CCGACAATACGAACGCCTTG	26
*Sr32*	csSr32#1	184	F-5’GGTTTGGTGGCAACTCAGGT R-5’ CATAAGCCAAAGAGGCACCA	27
*Sr38*	Xcmwg682	259	F-5’AGGGGCTACTGACCAAGGCT R-5’TGCAGCTACAGCAGTATGTACACAAAA	29

### Resistance determination

The whole cultivars (lines) were planted in 12 cm diameter porcelain pots. The Little Club (LC) was used as the control to ascertain the viability of spores or successful inoculation of races to cultivars. Leaves of 7-days-old seedlings were moistened by water with 0.1% Tween 20 using an atomizer, then sprayed 1 g of fresh urediospores and dried talc in a ratio of 1:20 (V:V). The inoculated seedlings were kept in a dew chamber for 16~20 h dark at 18~22°C and RH of 95% before transferring to a greenhouse adjusted at 18~22±1°C. Infection types (ITs) were assessed 14 days after inoculation using 0–4 IT scale described by Stakman et al. [23]. ITs were then grouped into two categories. ITs ‘0’, ‘;’, ‘1’, ‘1+’, ‘2’, ‘2+’ and X were considered as low infection types (resistance) while ITs ‘3-’, ‘3’, ‘3+’ and ‘4’ as high infection types (susceptible) [23].

## Results

### Wheat seedling resistance

The reaction of 119 main wheat cultivars in Yunnan to the *Pgt* races are shown in Table 3. Seventy-seven (64.7%) of the tested wheat varieties showed varying levels of resistance to races 21C3CTHTM, 34MKGSM and 34C3RKGQM. The remaining 42 wheat cultivars (35.3%) showed varying levels of susceptibility.

**Table 3:** Resistant proportion of 119 wheat cultivars to wheat stem rust.

Race	Susceptible		Resistance	
	Number of cultivars	Percentage/%	Number of cultivars	Percentage/%
34MKGSM	24	20.2	95	79.8
21C3CTHTM	22	18.5	97	81.5
34C3RKGQM	36	30.3	83	69.7
All tested races	77	64.7	42	35.3

A total of 97 cultivars showed different levels of resistance to race 21C3CTHTM, accounting for 81.5% of all the tested cultivars. Ninety-five cultivars were resistant to race 34MKGSM, and 24 showed susceptibility. A total of 83 cultivars showed resistance to 34C3RKGQM, accounting for 69.7% of all the tested cultivars, which was relatively low compared to resistance to 34MKGSM and 21C3CTHTM.

### *Sr25* screening

**Fig 1:**
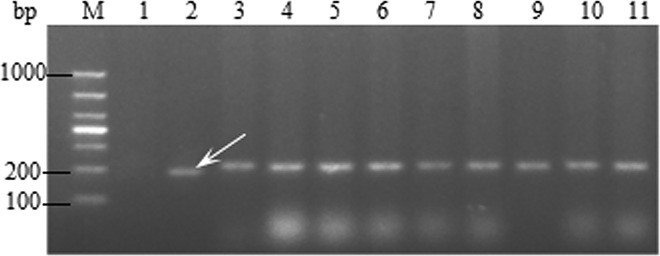
Amplification result for parts of wheat varieties with Xwmc221. Lane 1–11, Water control, Monogenic *Sr25*, Little Club, Kunmai 4, Kunmai 5, Jingmai 8, Jingmai 9, Jingmai 10, Jingmai 11, Jingmai 12, and Demai 3. ‘M’ indicates 1000 bp DNA ladder and white arrow indicates the position of the specific band.

LC was used as the negative control and the monogenic *Sr25* served as the positive control. A 190 bp specific band was amplified in the positive control using the primer Xwmc221. This result showed that no amplification of the 190 bp band in the 119 wheat cultivars of Yunnan Province (Fig 1).

### *Sr26* screening

**Fig 2:**
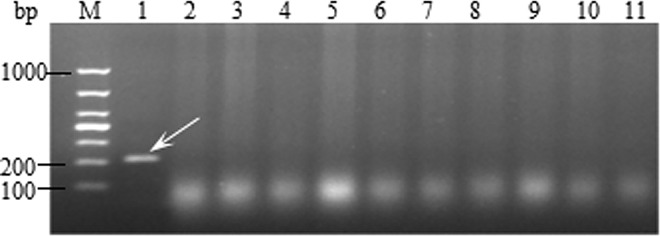
Amplification result for parts of wheat varieties with Sr26#43. Lane 1–11, Monogenic *Sr26*, Little Club, Water control, Jingmai 8, Jingmai 9, Jingmai 10, Jingmai 11, Jingmai 12, Jingmai 14, Jing 0202, and Jing 04–6. ‘M’ indicates 1000 bp DNA ladder and white arrow indicates the position of the specific band.

Mago et al. [24] developed a pair of RFLP markers for detection of wheat stem rust resistance gene *Sr26*, and the effectiveness of the specific marker *Sr26*#43 was evaluated. The results showed that the primer *Sr26*#43 amplified a 207 bp fragment, and was used to detect *Sr26* in the cultivars (Fig 2). LC was used as a negative control and monogenic *Sr26* was used as the positive control. Primer *Sr26*#43 amplified a 207 bp band in the positive control *Sr26*, while no bands were amplified in the remaining materials, indicating that the tested cultivars do not contain the resistance gene *Sr26*.

### *Sr28* screening

**Fig 3:**
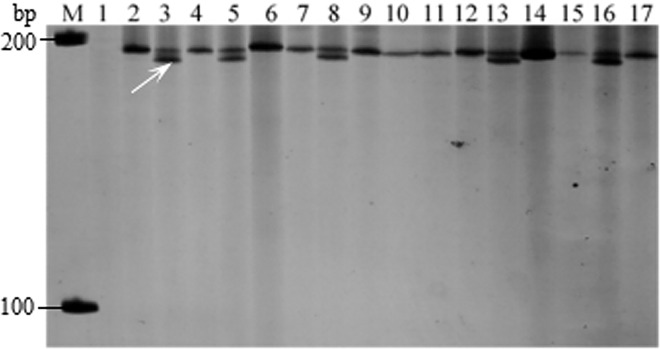
Amplification result for parts of wheat varieties with Wpt-7004. Lane 1–11, Water control, Little Club, Monogenic *Sr28*, Jing 04–6, Jing 0202, Jingmai 14, Jingmai 11, Jingmai 10, Jingmai 8, Yunza 7, Yunza 6, Yunxuan 2, Yunmai 54, Yunmai 53, Yunmai 52, Yunmai 47, and Yunmai 39. ‘M’ indicates 1000 bp DNA ladder and white arrow indicates the position of the specific band.

Rouse et al. [25] showed that the marker wPt-7004 amplified 194 bp and 166 bp fragments in the cultivars containing *Sr28* resistance genes. Further analysis confirmed that the amplification of the 194 bp fragment represented the specific band for *Sr28*. Polyacrylamide gel electrophoresis was used for detection of *Sr28*. As shown in Fig 3, the primer wPt-7004 amplified a band measuring 194 bp to 200 bp in the negative control, but an extra 194 bp band was amplified in the positive control *Sr28*. In this study, a similar 194 bp band was detected in Nanyuan 1, Feng 0103, Jing 07–2, Jingmai 10, Jing 05–1, 088–16, E33, Linmai 15, Jing 0202, Jingmai 10, Yunmai 54, Yunmai 47, and Feng 615, indicating that the 12 tested cultivars carried *Sr28* (Table 4).

**Table 4:** Results for wheat seedling resistance and molecular detection.

Cultivars/lines	21C3CTHTM	34MKGSM	34C3RKGQM	*Sr25*	*Sr26*	*Sr28*	*Sr31*	*Sr32*	*Sr38*
Yunmai 39	1^*^	;	1+	-	-	-	+	-	-
Yunmai 42	4	3	4	-	-	-	-	-	-
Yunmai 43	3	4	4	-	-	-	-	-	-
Yunmai 47	0	1	;	-	-	+	+	-	-
Yunmai 48	1+	4	3-	-	-	-	-	-	-
Yunmai 51	3+	3	3+	-	-	-	-	-	-
Yunmai 52	4	3	4	-	-	-	-	-	-
Yunmai 53	3+	;	3	-	-	-	-	-	-
Yunmai 54	;	0	1+	-	-	+	+	-	-
Yunmai 56	3	1+	1	-	-	-	-	-	-
Yunxuan 2	1	0	0	-	-	-	-	-	-
Yunxuan 3	0	0	0	-	-	-	-	-	-
Yunxuan 11–12	0	1	1+	-	-	-	+	-	-
Yunza 5	4	3	3+	-	-	-	-	-	-
Yunza 6	3	3	4	-	-	-	-	-	-
Yunza 7	3-	3-	4	-	-	-	-	-	-
Kun 022-222-1	0	;1-	1	-	-	-	+	-	+
Kunmai 4	0	;	0	-	-	-	-	-	-
Kunmai 5	0	0	0	-	-	-	+	-	-
Jingmai 8	0	0	;	-	-	-	+	-	-
Jingmai 9	0	0	1	-	-	-	+	-	-
Jingmai 10	0	0	0	-	-	+	+	-	-
Jingmai 11	0	1	;	-	-	-	+	-	-
Jingmai 12	0	0	0	-	-	-	+	-	-
Jingmai 14	0	0	1	-	-	-	+	-	+
Jing 0202	;	1	0	-	-	+	+	-	-
Jing 04–6	0	1	0	-	-	-	+	-	-
Demai 3	2	0	3	-	-	-	-	-	-
Demai 7	0	0	0	-	-	-	+	-	-
De 05–81	0	0	1	-	-	-	-	-	+
Linmai 6	1	3-	3	-	-	-	-	-	-
Linmai 15	0	0	0	-	-	+	+	-	-
Fengyin 03–2	0	1	3	-	-	-	-	-	-
Feng 05–394	;1-	0	;	-	-	-	+	-	-
Yunmai 101	0	;1-	0	-	-	-	+	-	-
Feng 1124	;	0	0	-	-	-	-	-	+
Yumai 1	0	0	0	-	-	-	+	-	-
Yumai 2	;1	;	1	-	-	-	+	-	-
Yumai 3	0	0	0	-	-	-	+	-	-
Fengmai 31	1	;	3-	-	-	-	-	-	-
Fengmai 32	;	0	1	-	-	-	+	-	-
Fengmai 33	;1-	;1	;	-	-	-	+	-	-
Wenmai 12	;	0	1+	-	-	-	+	-	-
Chumai 12	;1	1	;	-	-	-	+	-	-
Liangmai 4	0	0	0	-	-	-	+	-	+
Mian 1971–98	3	1	3	-	-	-	-	-	-
De 0716	0	0	0	-	-	-	+	-	-
Dianmai 34	0	0	3	-	-	-	-	-	-
Chu 2008 jian-4	0	0	;	-	-	-	+	-	-
91E001	0	1+	2	-	-	-	-	+	-
R101	;	0	0	-	-	-	-	-	-
R57	0	1	1	-	-	-	+	-	+
E33	0	0	0	-	-	+	+	-	-
06D_6_-6	1	0	1+	-	-	-	+	-	-
Yu 09–5	0	0	2	-	-	-	-	-	-
Feng 615	;	;1-	0	-	-	+	-	-	-
Yimai 1	3	4	4	-	-	-	-	-	-
Yixuan A03-2	;	1+	0	-	-	-	-	-	+
Yixi 96–6	0	4	4	-	-	-	-	-	-
Yixi 2003–64	0	3	4	-	-	-	-	-	-
Jing 05–1	0	0	0	-	-	+	+	-	-
Jing 07–2	1	0	1	-	-	+	-	-	-
Linmai 17	0	0	0	-	-	-	+	-	-
Chu 06–9	1	;	1	-	-	-	-	-	-
Feng 1128	0	1	0	-	-	-	-	-	+
SH 710	2	0	3	-	-	-	-	-	-
De 07–19	0	0	0	-	-	-	+	-	-
De 07–20	0	0	0	-	-	-	+	-	-
De 07–21	0	0	0	-	-	-	+	-	-
De 08–3	;	0	1	-	-	-	+	-	+
De 08–4	0	0	0	-	-	-	-	-	-
De 08–11	0	0	0	-	-	-	-	-	-
Wen 05–1	0	0	1+	-	-	-	-	-	-
Wen 06–3	0	0	0	-	-	-	-	-	-
084–12	3-	2	3+	-	-	-	-	-	-
Feng 0483	0	0	0	-	-	-	-	-	-
Feng 0103	1+	3	4	-	-	+	-	-	-
Feng 0230	0	;	0	-	-	-	-	-	-
Mosha	0	0	0	-	-	-	-	-	-
Jingxuan 9	0	0	0	-	-	-	-	-	-
Guoji 13	;	0	;	-	-	-	-	-	-
Yunmai 29	0	1	0	-	-	-	-	-	-
Mianyang 19	0	4	3	-	-	-	-	-	-
I_1_	3+	4	4	-	-	-	-	-	-
I_18_	0	0	1+	-	-	-	-	-	-
Fengmai 13	3	;1	4	-	-	-	-	-	-
Fengmai 24	1	1+	0	-	-	-	-	-	-
Jingmai 7	0	4	0	-	-	-	-	-	-
Demai 4	0	0	0	-	-	-	+	-	-
Nanyuan 1	1	4	3	-	-	+	-	-	-
Mianyang 20	3	4	4	-	-	-	-	-	-
Kunming chunmai	0	1+	0	-	-	-	-	-	-
Nanda 2419	3	4	4	-	-	-	-	-	-
Fengmai 34	0	0	0	-	-	-	-	-	-
Fengmai 35	4	0	4	-	-	-	-	-	-
Fengmai 36	0	0	1	-	-	-	-	-	-
Fengmai 37	0	0	3	-	-	-	-	-	-
Fengmai 38	0	0	0	-	-	-	-	-	-
Fengmai 39	0	0	0	-	-	-	-	-	-
Yimai line 2003–27	4	4	4	-	-	-	-	-	-
Yimai 10	0	4	4	-	-	-	-	-	-
K07-295	0	;	1	-	-	-	+	-	+
K042-39	1+	0	1	-	-	-	+	-	-
4–12	3+	1	2	-	-	-	-	-	-
4–8	0	0	0	-	-	-	-	-	-
05–1	;1-	0	3	-	-	-		-	-
088–16	0	0	;	-	-	+	+	-	-
09D_4_-1	0	1	0	-	-	-	+	-	-
09D_4_-6	4	4	1+	-	-	-	-	-	-
08 yu F-5	0	3-	0	-	-	-	-	-	-
066–3	0	0	1+	-	-	-	+	-	-
017–10	0	1	2	-	-	-	-	-	-
HX-06-1	0	0	4	-	-	-	-	-	-
098–2	3	0	0	-	-	-	-	-	-
098–4	0	0	0	-	-	-	-	-	-
098–6	1	0	2	-	-	-	-	-	-
098–10	3	3	4	-	-	-	-	-	-
098–12	1	;	3	-	-	-	-	-	-
098–13	0	1	3	-	-	-	-	-	-

*Infection types (ITs) are based on a 0-to-4 scale, ITs of 0,;, ; 1, 1, and 2 are indicative of a resistant (low) response and ITs of 3 or 4 are a susceptible (high) response.

### *Sr31* screening

**Fig 4:**
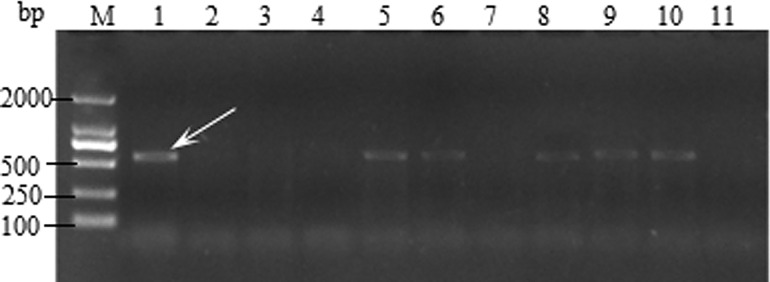
Amplification result for parts of wheat varieties with SCSS30.2_576_. Lane 1–11, Monogenic *Sr31*, Little Club, Water control, Fenyin 03–2, Feng 05–394, Yunmai 101, Feng 1124, Yumai 1, Yumai 2, Yumai 3, and Fengmai 31. ‘M’ indicates 2000 bp DNA ladder and white arrow indicates the position of the specific band.

The SCSS30.2_576_ marker is linked to stem rust resistance gene *Sr31*. The *Sr31* genes transferred from rye into wheat (*Triticum aestivum* L.) contributes to resistance in all the virulent pathotypes of stem rust (*P*. *graminis* f. sp. *tritici*) found in China. The SCSS30.2_576_ marker amplified an expected PCR product of approximately 576 bp [26] in the positive control *Sr31* (Fig 4). It acted as a specific marker with no product generated in lines that do not carry *Sr31*. Among the tested cultivars, 43 cultivars tested positive for the *Sr31* marker (Table 4).

### *Sr32* screening

**Fig 5:**
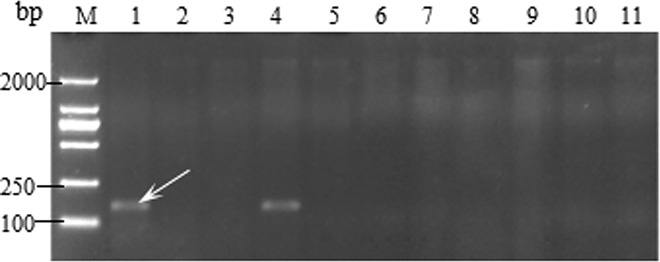
Amplification result for parts of wheat varieties with csSr32#1. Lane 1–11, Monogenic *Sr32*, Little Club, Water control, 91E001, R101, R57, E33, 06D_6_-6, Yu 09–5, Feng 615, and Yimai 1. ‘M’ indicates 2000 bp DNA ladder and white arrow indicates the position of the specific band.

*Sr32* is located on the short arm of chromosome in Sears’ wheat *Aegilops speltoides* 2D-2S#1 translocation line C82.2 [27]. A dominant marker, cs*Sr32*#1, derived from an AFLP fragment [27] amplified 184bp PCR product in wheat lines carrying *Sr32*. In this study, the marker cs*Sr32*#1 was used to determine the presence of *Sr32* in 119 wheat materials. The band was amplified only in the positive control *Sr32* and one wheat cultivar 91E001, but none was detected in the remaining cultivars (Fig 5).

### *Sr38* screening

**Fig 6:**
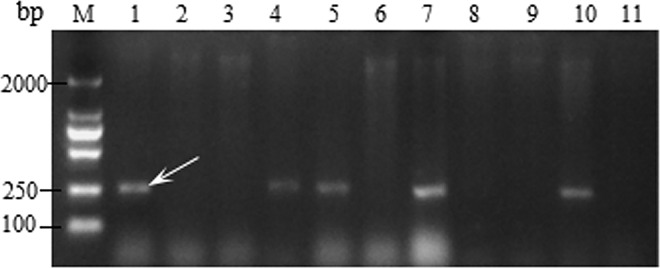
Amplification result for parts of wheat varieties with Xcmwg682. Lane 1–11, Monogenic *Sr38*, Little Club, Water control, De 05–81, Feng 1124, Yumai 1, Liangmai 4, De 0716, Dianmai 34, Feng 1128, and De 07–19. ‘M’ indicates 2000 bp DNA ladder and white arrow indicates the position of the specific band.

Rust resistance genes *Lr37*, *Sr38*, and *Yr17* are located within a segment of *T*. *ventricosum* (Tausch) Cess. chromosome 2NS translocated to the short arm of bread wheat chromosome 2AS [28]. Helguera et al. [29] developed polymerase chain reaction (PCR) assays based on RFLP marker cMWG682 to facilitate the transfer of this cluster of rust resistance genes into commercial wheat (*T*. *aestivum* L.) cultivars. The marker played a major role in molecular assisted breeding (MAS) [30]. Li et al. [31] validated the marker cMWG682 using 126 wheat lines and indicated that cMWG682 amplified a 259 bp fragment in the positive control Yr17/6*Avocet S and 12 cultivars. In this study, monogenic *Sr38* was used as a positive control and LC as a negative control. A 259 bp fragment was amplified in the positive control *Sr38* and 10 of the tested wheat cultivars, using primer cMWG682 (Fig 6), suggesting that the 10 cultivars (lines) carried *Lr37*-*Sr38*-*Yr17* (Table 4).

## Discussion

Gene *Sr25* transfer to wheat *Thinpyrum ponticum*, occurs on the long arm of chromosome 7DL. It is usually closely linked to leaf rust resistance gene *Lr19*. Another *Th*. *ponticum-*derived gene resulted in undesirable yellow flour [32], and improved the yield [21]. The *Sr25* resistance is affected by growth periods and temperature. The resistance in seedling stage is higher than in adult stage and is more easily susceptible under high temperature conditions [33]. Due to its resistance to Ug99, the specific primer Xwmc221 was used for detection of 150 cultivars in China, but no *Sr25* was identified [21]. In this study, the same primer pair was used for detection of *Sr25* in119 wheat cultivars (lines) from Yunnan Province. The results also indicated that none of the tested wheat cultivars contained the gene. Therefore, the gene was targeted in the future breeding programs to improve the resistance level of wheat cultivars to Ug99 in China.

The wheat stem rust resistance gene *Sr26* (derived from *Agropyron elongatum*) is derived from foreign chromosome translocated to wheat chromosomes [24]. This gene located on chromosome 6AL, confers resistance to all races of wheat stem rust worldwide [9] and is widely used in Australia [24]. It is reported that the Chinese wheat varieties may contain *Sr26*. Two out of 448 wheat cultivars contain *Sr26* that was identified by researchers [34–36]. In this study, the Yunnan varieties were tested for presence of *Sr26*. However, the gene was not found in any of the materials tested, which indicated that Yunnan wheat varieties lack the *Sr26*.

*Sr28* originates in *T*. *aestivum* L. Kota. Kota has been used as a differential host by Stakman since 1962. However, due to ineffectiveness to most races of *Pgt*, its application was limited in resistance breeding [25]. With the emergence of Ug99, researchers focused on the discovery of genes or gene combinations resistant to Ug99, to strengthen the resistant cultivars in the international wheat stem rust nursery of Kenya. SD1691 resistant to Ug99 (TTKSK) was studied by Rouse et al. [37], and found to harbor the *Sr28* resistance gene [25]. In addition, *Sr28* was ineffective to several popular American races of *Pgt*. However, it was combined with other resistance genes to yield effective resistance gene combinations, and has been used in breeding disease resistance [25]. Therefore, the diversity arrays technology (DArT) [38] marker closely linked to *Sr28* was developed for more convenient screening and identification of the effective gene. Similarly, *Sr28* was also ineffective to many *Pgt* races in China [39, 40]. Chen et al. [35] reported that Chinese wheat cultivars carry *Sr28*. In this study, the DArTmarker wPt-7004 screened by Rouse was used to detect the gene in Yunnan wheat cultivars (lines). The results showed that 12 wheat varieties (lines) carried this gene. These were Nanyuan 1, Feng 0103, Jing07-2, Jing 05–1,088–16, E33, Linmai 15, Jing 0202, Jingmai 10, Yunmai 54, Yunmai 47, and Feng 615.

The gene *Sr31*, which confers a high degree of effectiveness to stem rust was introgressed into bread wheat from ‘Petkus’ rye as a 1BL/1RS translocation [26]. *Sr31* has been widely used in Chinese and global wheat stem rust breeding programs [26, 41, 42]. However, wheat stem rust has re-emerged as a major threat to global wheat production following the emergence of the new race Ug99 virulent to *Sr31* in 1999 [5]. To date, no virulent race against *Sr31* has been reported in China [41]. In our study, 43 cultivars (lines) probably carried *Sr31*. Because *Sr31* has been widely used in Chinese wheat breeding since 1970s, and identified that many cultivars carry this gene [41, 42]. Our results were consistent with previous reports. For example, Wang et al. [43] and Zhang et al. [44] identified a 1BL/1RS translocation in 211 and 75 wheat cultivars (lines) using the co-dominant PCR marker. Their results showed that 81 and 25 wheat varieties carried this gene, respectively. Conversely, pedigree tracking indicated that resistant materials carrying *Sr31* such as ‘Kavkaz’ and ‘Luofu’ lines were widely used in wheat breeding in Yunnan Province [45], suggesting the origin of resistance genes in these wheat varieties. In addition, a specific band was amplified in Yunmai 51 (91B-831/92B-84) and Fengmai 31 (918M40-1) using SCSS30.2_576_ marker. However, the two cultivars were highly susceptible to *Pgt* in China and no race virulent to *Sr31* was found, suggesting that the two cultivars do not contain the gene *Sr31*. This finding may be attributed to false-positive results, which are common challenges associated with MAS. For example, Yu et al. reported that the variety Thatcher does not carry *Sr2* but tested positive [46].

*Sr32* present on the short arm of chromosome 2S#1 was transferred to wheat from *Aegilops speltoides* Tausch [47]. *Sr32* exhibited strong resistance against the predominant race group 21C3 in China [13, 39]. In this study, we found that 91E001 carried *Sr32*. The wheat cultivar 91E001 used in breeding programs was introduced from Mexico to China, with strong resistance against wheat stripe rust [48]. Additionally, *Sr32* was shown to be effective against seven race variants of Ug99 lineage [9], and was used in future resistance breeding. The durability of resistance genes is enhanced by deploying pyramids in cultivars.

The translocation line VPM1 of *Triticum aestivum* L. containing 2NS chromosome segments (25~38cM) from *Ae*. *ventricosa* was cultivated by Maia in 1967 [32]. The fragment carried three effective genes against rust: *Lr37* against leaf rust, *Sr38* against stem rust and *Yr17* against stripe rust. These three genes have been widely used by breeders worldwide [32, 41]. *Sr38* is no longer resistant to new races related to Ug99. No virulent *Pgt* race to *Sr38* has been found in China. PCR analysis was used for effective screening of resistance genes *Lr37*-*Yr17*-*Sr38* established by Helguera [29], which played an important role in MAS [30], and was used in this study for identification of genes in wheat cultivars of Yunnan Province. The results showed 10 wheat cultivars harboring the gene. The resistance of these cultivars against the Chinese races 34MKGSM and 21C3CTHTM may be attributed to the *Lr37*-*Yr17*-*Sr38* genes.

In this study, the molecular genetic markers of *Sr25*, *Sr26*, *Sr28*, *Sr31*, *Sr32* and *Sr38* in wheat cultivars against *Pgt* races were used to identify the 119 wheat cultivars (lines) in Yunnan province. Forty three and 10 wheat cultivars (lines) are likely to carry *Sr31* and *Sr38*, respectively. *Sr31* and *Sr38* have been widely used in Chinese stem rust resistance breeding [41]. Although *Sr31* and *Sr38* are no longer resistant to new races related to Ug99, they are still useful when combined or used as pyramids with other genes effective against Ug99. Overall, wheat cultivars in Yunnan province lack the genes *Sr25* and *Sr26*, which are effective against Ug99. *Sr28* alone was detected in 12 wheat cultivars including Nanyuan 1, Feng 0103, Jing 07–2, Jing 05–1, 088–16, E33, Linmai 15, Jing 0202, Jingmai 10, Yunmai 54, Yunmai 47, and Feng 615. *Sr32* was detected in one cultivar 91E001. Spread of Ug99 into China will most likely lead to colonization of the southwest area such as Yunnan and Sichuan provinces, where *Pgt* is already prevalent [41]. Therefore, the widespread cultivation of these wheat cultivars susceptible to Ug99 in Yunnan greatly increases the risk of its epidemic. Fortunately, the two genes *Sr28* and *Sr32* might play a role in the prevention of colonization and invasion of Ug99. The 12 cultivars containing *Sr28* and the 91E001 carrying *Sr32* identified in this study will be useful for resistance breeding and propagation of cultivars.
